# Micro Electrochemical Milling of Micro Metal Parts with Rotating Ultrasonic Electrode

**DOI:** 10.3390/s20226617

**Published:** 2020-11-19

**Authors:** Yong Liu, Haoran Chen, Shenghai Wang, Kan Wang, Minghao Li, Tengfei Peng

**Affiliations:** School of Mechanical, Electrical & Information Engineering, Shandong University, Weihai 264209, China; rzliuyong@163.com (Y.L.); 201816473@mail.sdu.edu.cn (H.C.); wangkan@sdu.edu.cn (K.W.); 201816477@mail.sdu.edu.cn (M.L.); 201936600@mail.sdu.edu.cn (T.P.)

**Keywords:** micro electrochemical milling, ultrasonic vibration, surface quality, machining localization, microstructures

## Abstract

With the rapid development of MEMS, the demand for metal microstructure is increasing. Micro electrochemical milling technology (MECM) is capable of manufacturing micro metallic devices or components based on the principle of electrochemical anode dissolution. To improve the capacity of MECM, this paper presents a compound method named ultrasonic vibration-assisted micro electrochemical milling technology (UA-MECM). Firstly, the simulation and mathematical model of UA-MECM process is established to explain the mechanism of ultrasonic vibration on micro electrochemical milling. Then, the effects of ultrasonic parameters, electrical parameters and feedrate on machining localization and surface quality are discussed considering sets of experiments. The surface roughness was effectively reduced from *R*a 0.83 to *R*a 0.26 µm with the addition of ultrasonic vibration. It turns out that ultrasonic vibration can obviously improve machining precision, efficiency and quality. Finally, two- and three-dimensional microstructures with good surface quality were successful fabricated. It shows that ultrasonic vibration-assisted electrochemical milling technology has excellent machining performance, which has potential and broad industrial application prospects.

## 1. Introduction

Intelligent micro-components have become a trend and play an important part in many demanding fields, especially in aeronautics, energy battery, medicine, etc. Micro-Electro-Mechanical-Systems (MEMS) were first applied in the field of aeronautics because of their high functional-density ratio [1,2]. This also poses a challenge to machining technology. The traditional material removal machining methods mostly uses heat removal machining, which can easily produce a thermal stress layer on the machined surface. Electrochemical micromachining (ECM) is a method for electrochemical etching of the surface of metal workpiece in the form of ions, so that the machining size can reach the micro range. Compared with traditional machining methods, the machined material is dissolved in the form of ions with theoretical processing accuracy up to nanometer scale. Because the electrode does not contact the workpiece in ECM, there is no residual mechanical stress, thermal stress and tool loss, and it is not limited by material hardness [3]. With the increasing requirements for the size and machining precision of micro-miniature components and difficult-to-cut materials, micro electrochemical machining has developed rapidly into the micro machining field, which has advantages of high machining precision, high surface quality and high machining efficiency [4]. Microgrooves and complex cavities are common machined structures in the micro electrochemical field. However, as the machined size decreases, especially for the large aspect ratio, the electrolyte solution cannot be discharged in time due to the small machining gap, leading to lower etching rate and being easy to short circuit. The electric field and flow field are distorted, and the machining accuracy is reduced because of the poor machining environment. To improve the localization of electrochemical milling machining, it is necessary to combine auxiliary methods to promote electrolyte circulation. High-speed rotating spiral electrode and vibration assistance are effective methods to improve electrolyte circulation, and the latter has become a research hotspot in the field of micro electrochemical machining.

Initially, ECM had low localization and stray corrosion, which seriously affects the quality of machining. However, the introduction of high-frequency pulse power supply makes micro electrochemical machining have high machining accuracy, surface quality and machining efficiency. In 2000, Schuster first applied ultrashort pulses to electrochemical machining between a tool electrode and a workpiece, improving localization of machining. He used 10-µm diameter cylindrical Pt electrode to machine a three-dimensional micro cavity convex platform structure with te largest border size of 40 µm [5]. Liu applied ultrashort pulse power supply to greatly improve the localization of micro electrochemical machining and analyzed the influence of pulse width on machining shape accuracy and the influence of electrode working end shape on surface quality [6]. Hu et al. firstly adopted a 704-silica material to fabricate the side-insulation film on the tool electrode to isolated from electrolyte, and some microstructures with vertical sidewall were successfully machined as a result [7]. Rathod et al. used sidewall insulation electrode reduced the width overcut of microgroove by 58.64% and reduced the taper angle from 58.39° to 25.20° for microgroove [8]. Rathod et al. successfully milled a variety of different cross-sections by using disk-shaped electrodes and adjusting the pulse voltage duty ratio in the process [9]. Liu et al. established the layered milling model and machined 2D and 3D complex structures with good shape precision [10]. Mishra et al. finished machining with good surface finish using rotating electrode on nickel-based alloy [11]. Liu and Qu analyzed the anodic polarization curves of TB6 titanium alloy in NaNO_3_ solution and grooves and flat surfaces having uniform machined surfaces were fabricated successfully by electrochemical milling [12]. Zhou fabricated high-quality microgrooves arrays on phosphor bronze surface by through-mask electrochemical micromachining with high inlet pressure and low pulse duty cycle. A surface covered by microgrooves increased the friction coefficient from 0.245 to 0.334 [13]. He et al. proposed the method of electrochemical machining of micro slots by using shaped electrode instead of cylindrical electrode. The results show that the shaped electrode can not only improve the machining speed, but also reduce the side gap and frontal gap of the micro slots [14]. Chen et al. put forward a double nozzle liquid supply mode to reduce the geometric deviation such as the fillet and taper of the left- and right-side walls of the groove [15]. To improve the flatness of workpiece, Yue et al. proposed a new tool substitute with additional bottom outlet holes. The tool electrode can significantly improve the flatness and surface roughness of the workpiece [16]. Lei et al. proposed to eliminate the step effect of stacked three-dimensional (3D) microelectrodes by using layered scanning micro electrochemical machining. The results show that the step effect of 3D microelectrode was eliminated when the voltage was 4.0 V, the offset distance was 200 µm and the layer thickness was 20 µm [17]. Zeng et al. studied the combined milling machine of micro-discharge machining (EDM) and ECM, which used different media with the same electrode on the same machine tool. Because ECM eliminated the recast layer and surface defects caused by EDM, the size and shape accuracy of the workpiece machined by combined milling machine were accurately controlled, which was much better than that of only using micro ECM [18]. Liu et al. proposed a method combining electrochemical milling with grinding, in which two types of electrodes were used in different processing stages. First, electrochemical milling was performed, and then 27% of the groove depth was removed by electrochemical grinding. The surface roughness decreased from Ra 4.853 to 0.372 [19].

To improve the localization of electrochemical micromachining, it is necessary to combine auxiliary methods to promote electrolyte renewal. Vibration assistance is an effective method to improve electrolyte renewal, and it is also a research hotspot in the field of micro electrochemical machining. ultrasonic vibration assistance is helpful to remove reaction products and heat in the machining zone, which is conducive to material diffusion, minimizes passivation, provides the best hydrodynamic conditions, improves the depth to width ratio of the machined size and affects the electrolysis reaction through sonochemical reaction [20].

Numerous scholars have done a lot of research on ultrasonic vibration-assisted electrochemical micromachining and achieved some results. Hewidy et al. found that low frequency vibration of tool electrodes periodically changes the pressure in the micro electrochemical machining gap, which promotes the discharge of electrolyte products and the renewal of fresh electrolyte, enhances the circulation of electrolyte through the interface, permits the use of higher current density and improves the surface machining quality [21]. Natsu et al. studied the influence of the direction and amplitude of ultrasonic vibration. The results show that the combined vibration of horizontal and vertical direction is more beneficial to improve the machining speed and shape accuracy [22]. Mitchell-Smith et al. found that ultrasonic vibration was beneficial to remove the passive film formed in the electro-hydraulic micro electrochemical machining process, and it improved the machining stability and aspect ratio. Under the same optimized machining parameters, the machined surface roughness decreased from 0.245 to 0.168 µm [23]. Fang et al. verified that vibration effectively promotes the discharge of bubbles by high-speed video camera, thereby improving machining efficiency and machining quality [24,25,26,27]. Goel and Pandey studied the effects of various machining parameters on material removal rate and hole taper in electrode ultrasonic vibration-assisted micro electrochemical drilling. The results show that ultrasonic vibration is good for material removal and the perpendicularity of hole [28]. Wu et al. improved the surface quality and machining depth of the machined microcavity through the vibration feed of cathode electrode and the combination of micro electrochemical machining and electropolishing. At the same time, adding B_4_C particles to the electrolyte can remove the electrochemical reaction attachment on the electrode surface and improve machining effect [29]. Li et al. proposed an ultrasonic-assisted pulse electrochemical grinding method. The influence of vibration amplitude on grinding force, material removal rate and workpiece surface roughness under different machining parameters such as applied voltage and rotating speed was studied [30].

However, the current research on ultrasonic vibration-assisted micro-electrochemical machining is not systematic and deep enough, especially on ultrasonic vibration-assisted micro electrochemical milling [31]. Therefore, aiming at the gap in the research, this paper systematically studies the effects of various parameters by combining ultrasonic vibration with high-speed rotating spiral electrode. Three-dimensional structures with smaller surface roughness were machined by optimizing the machining parameters.

## 2. Materials and Methods

**Figure 1:**
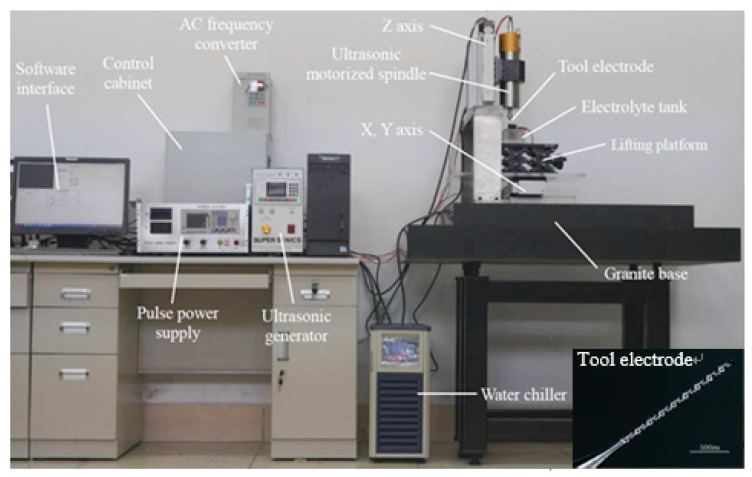
Experimental setup for UA-MECM.

In this paper, the cathode is a spiral cylindrical electrode with diameter of 100 µm, the anode is SUS304 with thickness of 510 µm and the electrolyte is NaNO_3_ with mass fraction of 5%. The spiral electrode is clamped to the front end of the ultrasonic spindle by a collet chuck, and the ultrasonic motorized spindle drives the tool electrode for ultrasonic vibration and high-speed rotation. Figure 1 is the experimental setup for UA-MECM.

**Figure 2:**
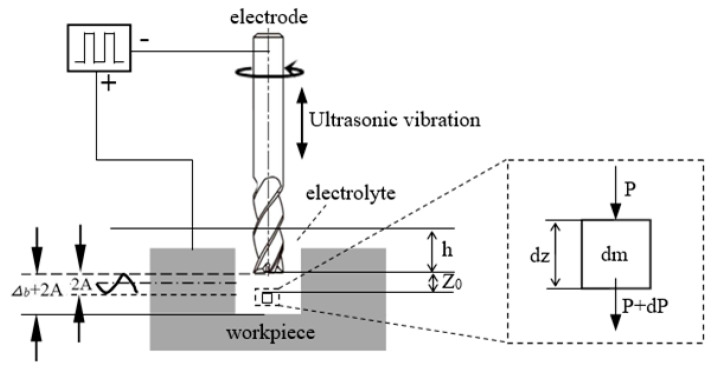
Schematic diagram of UA-Micro electrochemical machining gap.

During the machining process, the tool electrode makes periodic vibrations in the vertical direction, as shown in Figure 2. The displacement of tool *z* can be simplified to sinusoidal motion:

(1) $$z=A\sin[\omega \left(t-\frac{z_{0}}{c}\right)]$$

where *ω* is the vibration frequency and *c* is the ultrasonic velocity.

There is a pressure unit hypothetically, as shown in Figure 2, and, according to Newton’s second law, the pressure at any point in the *z* direction in the inter-electrode gap can be expressed as:

(2) $$P=\rho g(h+z)+\omega Ac\rho \cos\omega (t-\frac{z}{c})+P_{0}$$

where *ρ* is the density of electrolyte, *g* is gravity acceleration, *h* is the distance from electrolyte surface to the end of electrode and *P*_0_ is the atmospheric pressure.

**Figure 3:**
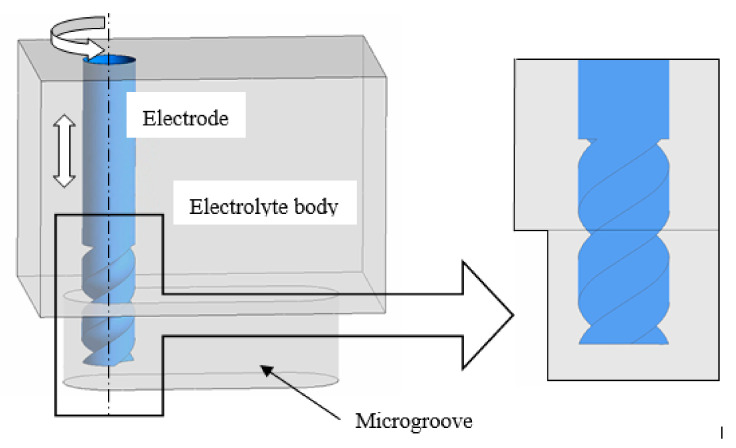
Flow field model in the machining gap.

**Figure 4:**
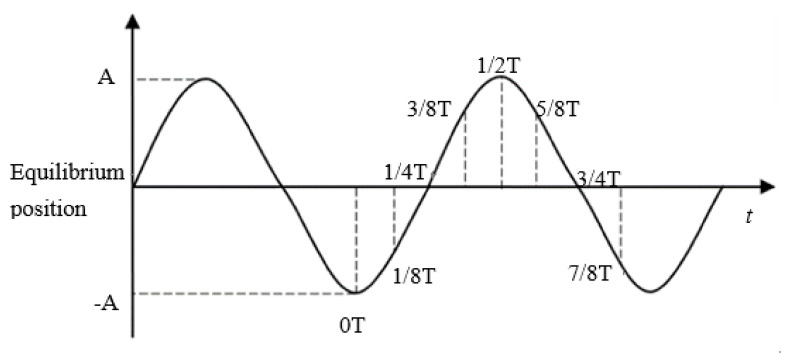
Ultrasonic vibration sinusoidal motion.

Equation (2) shows that the pressure in the narrow machining gap changes periodically when tool electrode is vibrated with ultrasonic frequency. To visually implicate the phenomenon, a simulation was conducted, as shown in Figure 3, and the simulation parameters are shown in Table 1. The ultrasonic vibration of the tool electrode can be simplified to sinusoidal motion, as shown in Figure 4. When setting the boundary conditions, the ultrasonic vibration of the electrode boundary is controlled by UDF. The program language of ultrasonic vibration velocity is defined as:

(3) vel=0.5×3.14×sin(50,000×3.14×time)

**Figure 5:**
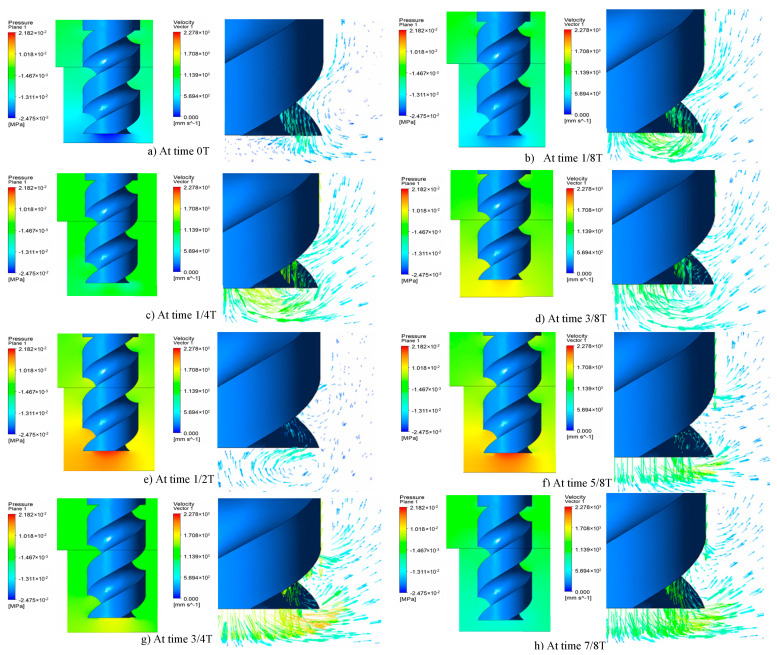
Simulation diagram of electrolyte circulation.

Figure 5a–e shows that, when the electrode moves upward from the lowest position, a negative pressure zone is formed at the end of the electrode and the electrolyte is pressed into the machining gap under the action of atmospheric pressure. A positive pressure zone begins to generate at the end of the electrode as the electrode reaches the peak position. When the electrode moves downward, as shown in Figure 5 f–h, the positive pressure zone squeezes the electrolytic fluid out of the machining gap. The rapid change of pressure happens periodically on the bottom of electrode during the machining process under the action of ultrasonic vibration. Therefore, the circulation of electrolyte is further promoted.

Due to the vibration of cathode electrodes in ultrasonic frequency, according to Faraday’s law [10], the material removal rate assisted by ultrasonic vibration *v* is as follows:

(4) $$v=\eta \omega_{1}\kappa E=\eta \omega_{1}\kappa_{0}{\left(1-\alpha \right)}^{m}\frac{u-E_{R}}{\left(\Delta b+A+A\sin(\omega t+\varphi )\right)}$$

where *η* is the current efficiency, *ω*_1_ is the electrochemical equivalent, *κ*_0_ is the electrolyte conductivity, *u* is the applied voltage, *E_R_* is the resolve voltage of anode, (1 − *α*)*^m^* is the conductivity correction factor, *α* is the hydrogen void ratio, *m* is a constant and Δ*_b_* is the balanced machining gap.

The hydrogen generated by the cathode gradually accumulates and adheres to the cathode surface and forms bubbles in the process of micro electrochemical machining without ultrasonic vibration, which hinders the electrochemical reaction and affects the machining precision. When tool electrode is vibrated in ultrasonic frequency, hydrogen cannot accumulate on the surface of the electrode to form large bubbles, and the bubbles are quickly discharged from the machining gap due to the influence of vibration and faster electrolyte circulation rate [32].

When tool electrode vibrates in ultrasonic frequency, the cavitation bubbles are generated and broken continuously during the change of pressure between positive and negative pressure. In the rupturing instant of cavitation bubble, high pressure, shock wave and micro-jet are generated, which accelerates the diffusion of ions and the renewal rate of electrolyte, reduces the potential of electrode surface, reduces the polarization of electrode and improves the conductivity of electrolyte. At the same time, the force produced by the breakdown of cavitation bubbles plays a finishing role on the machined surface and reduces the roughness. Therefore, the above theoretical analysis fully illustrates the feasibility of ultrasonic vibration assisted micro electrochemical machining.

**Figure 6:**
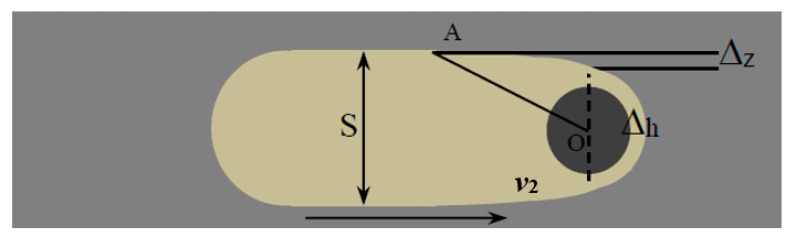
Simplified schematic diagram of milling process.

**Figure 7:**
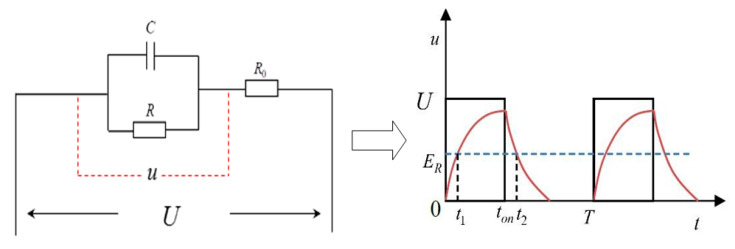
Equivalent circuit.

Figure 6 is the simplified model of milling process. The diameter of the electrode is d, the milling speed is *v*_2_, the machining gap is Δ*_h_*, the final groove width is *S*, the groove width increment caused by secondary corrosion is Δ*_z_* and Point A is the farthest position that the electrodes can influence. According to the double layer theory, the electrochemical reaction of machining area is driven by the potential difference on the double electric layer. To simplify the process, the equivalent circuit between the cathode and the anode in the electrolyte is shown in Figure 7 when using pulse power, and *C* is the equivalent capacitance, *R* is the resistance for machining area, *R*_0_ is the total resistance in circuits, *u* is the voltage across the machining area and *U* is input voltage of pulse power supply. The potential on the workpiece surface at a single pulse of the power supply does not rise instantly but has a certain delay. When the voltage is larger than the resolve voltage *E_R_*, the electrochemical reaction occurs. Therefore, it is assumed that the starting time and the ending time of electrochemical etching in a single pulse period are *t*_1_ and *t*_2_, respectively, and *u* can be expressed as:

(5) $$u=\left\{ \begin{array}{l} UB(1-e^{-\frac{t}{\tau }})\;(0\leq t<t_{\mathrm{on}})\\ UB(1-e^{-\frac{t_{\mathrm{on}}}{\tau }})e^{-\frac{t}{\tau }}\;(t_{\mathrm{on}}\leq t<t_{\mathrm{on}}+t_{\mathrm{off}}) \end{array}\right.$$

where *t*_on_ is the discharge time of single pulse periodic, *t*_off_ is the non-discharge time of single pulse period, *B* = *R*/(*R*+*R*_0_) and *τ* = *RR*_0_ *C*/(*R*+*R*_0_).

When the material removal speed *v* and feed rate *v*_2_ are approximately the same, the balance state is reached, and the side gap Δ*_h_* can be expressed:

(6) $$\begin{array}{l} {\bar{\Delta }}_{h}=\frac{\eta \omega_{1}\kappa_{0}{\left(1-\alpha \right)}^{m}(UB-E_{R})}{v_{2}T}t_{on}+\frac{\eta \omega_{1}\kappa_{0}{\left(1-\alpha \right)}^{m}\tau }{v_{2}T}[(UB-E_{R})\ln(1-\frac{E_{R}}{UB})\\ +E_{R}\ln\frac{E_{R}}{UB}-E_{R}\ln(1-e^{-\frac{t_{on}}{\tau }})] \end{array}$$

where *T* is the pulse period and *v_2_* is the milling feed. Due to the small amount of secondary corrosion during milling, Δ*_z_* can be ignored and the width of microgroove in ultrasonic vibration assisted micro electrochemical milling machining is as follows:

(7) $$\begin{array}{l} S=d+2\frac{\eta \omega_{1}\kappa_{0}{\left(1-\alpha \right)}^{m}(UB-E_{R})}{v_{2}T}t_{on}+2\frac{\eta \omega_{1}\kappa_{0}{\left(1-\alpha \right)}^{m}\tau }{v_{2}T}[(UB-E_{R})\ln(1-\frac{E_{R}}{UB})\\ +E_{R}\ln\frac{E_{R}}{UB}-E_{R}\ln(1-e^{-\frac{t_{on}}{\tau }})] \end{array}$$

Thus, the width of microgroove *S* is related to applied voltage *U*, pulse period *T*, pulse width *t*_on_ and milling speed *v*_2_. In the experiment, we used a single variable to verify its effect on groove width, respectively. From Equation (7), and bringing in the corresponding proportion, we can get the theoretical calculation values and compare them with the experimental values.

## 3. Discussion

The effects of ultrasonic parameters, electrical parameters and feed parameters on machining quality of micro electrochemical milling were verified by experiments, and the experimental parameters are shown in Table 2. To obtain the optimal parameters accurately, the key machining parameters were studied by the following sets of experiments.

### 3.1. Effect of Ultrasonic Amplitude on Machining Quality

Experiments were carried out under different amplitudes ranging from 0 to 10 µm. The speed of the ultrasonic spindle was 12,000 r/min, the milling feed rate was 0.8 µm/s, the pulse period was 5 µs, the pulse width was 0.5 µs and the pulse voltage was 6 V.

**Figure 8:**
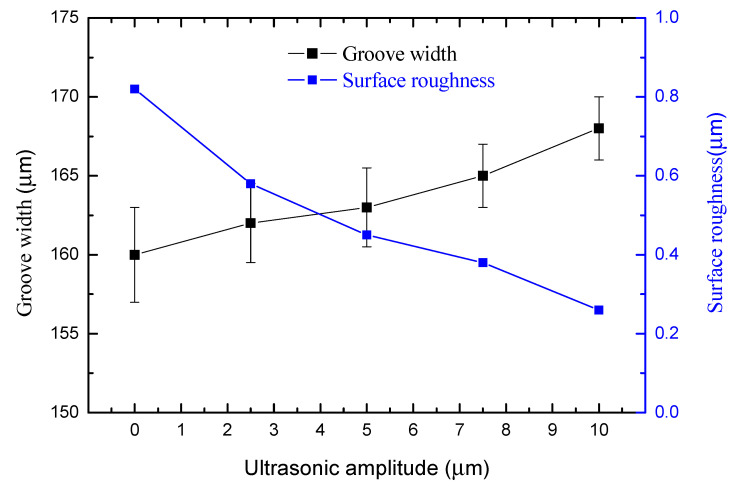
Change of groove width and surface roughness with ultrasonic amplitude.

**Figure 9:**
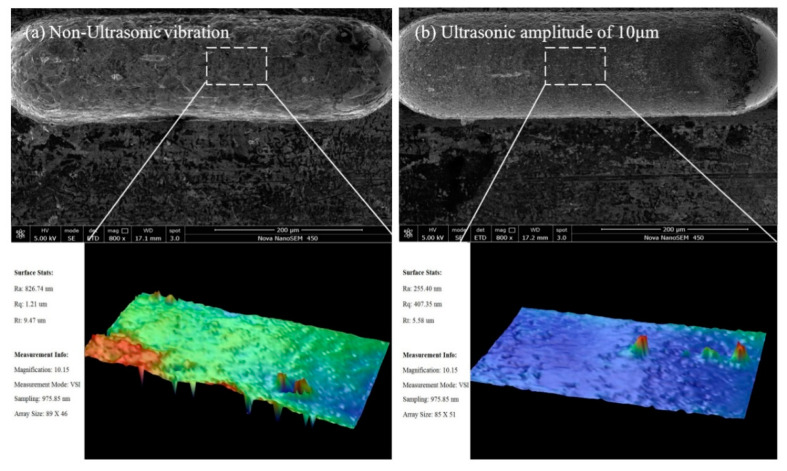
Comparison of surface roughness: (**a**) non-ultrasonic vibration; and (**b**) ultrasonic vibration of 10 µm.

Figure 8 shows that the groove width increases and the surface roughness decreases with the amplitude. The width of the microgroove machined without ultrasonic vibration assistance is 160 µm, and the width of the microgroove machined by 10-µm-amplitude ultrasonic vibration assistance is 168 µm when the other parameters are kept constant. Figure 9 is the comparison of surface roughness of microgrooves machined with the amplitude of 0 and 10 µm measured by field emission scanning electron microscope (Nova NanoSEM450, FEI Company, Hillsborough, OR, USA) and optical profilometer (Wyko NT9300, Veeco Instruments Inc., San Jose, CA, USA). The surface roughness machined without ultrasonic vibration is *R*a 0.82 µm, and the bottom and edge of the microgroove are not smooth. The surface roughness machined with the amplitude of 10 µm is *R*a 0.26 µm, and the surface machining quality and the shape accuracy of the microgroove are higher.

The above comparison results prove that the ultrasonic assistance improves the machining quality and reduces the surface roughness. The cavitation caused by ultrasonic vibration causes a moment when the cavitation bubbles near the electrode break up, resulting in the high-pressure shockwave and micro-jet. These changes accelerate the electrolyte circulation, making the charge move faster. As a result, the overpotential on the electrode surface and polarization are reduced. The machining gap is not blocked by the electrolytic products, so the corrosion electric field can work better on the workpiece. The flow field and electric field in the machining gap are in a more stable state. At the same time, the high pressure and micro-jet produced by the cavitation bubble have an effect of polishing the machined surface. High speed washing makes the electrolytic products unable to adhere to the surface, so the machined surface will be more uniform and smoother. Therefore, under the same machining parameters, the larger is the ultrasonic amplitude, the faster is the etching rate, the wider is the microgroove width and the higher is the machining quality of the microgrooves. To ensure the machining precision, the higher amplitude should be used, and the following experiments only used the amplitudes of 10 and 0 µm to embody the advantages of ultrasound vibration-assisted micro electrochemical milling.

The following group of experimental results show that the change trend of key machining factors is roughly consistent with the calculation results of mathematical model. However, there are still some differences, and the fitting is not very accurate. The main reason is that the mathematical model simplifies the actual working conditions but does not consider the influence of the dissolution characteristics of materials, temperature changes, electrolyte concentration gradient and other factors on the actual processing results.

### 3.2. Effect of Machining Voltage on Machining Quality

**Figure 10:**
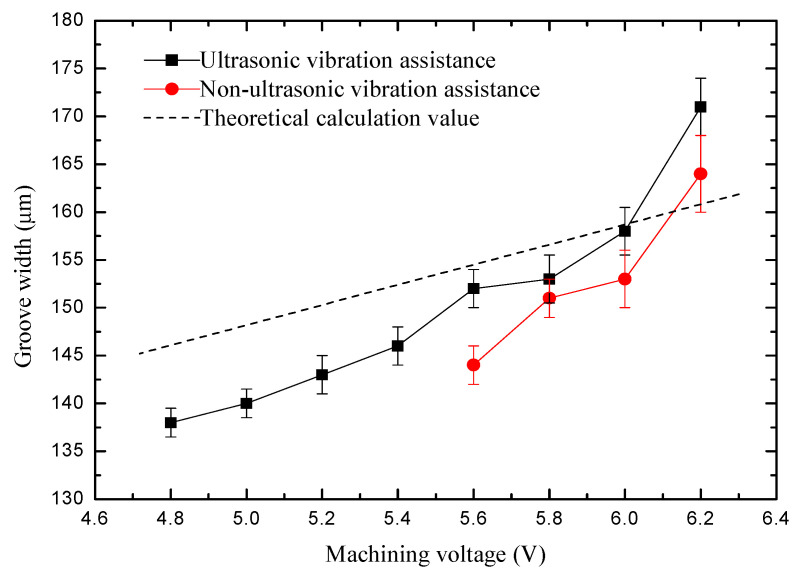
Change of groove width with machining voltage.

**Figure 11:**
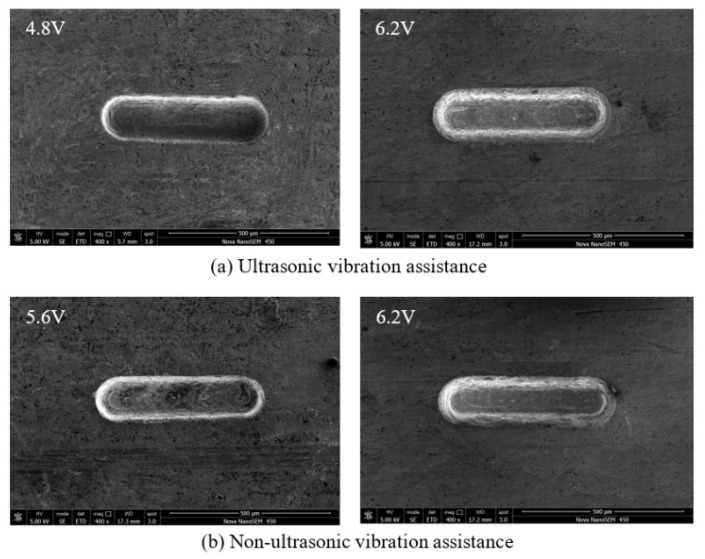
SEM micrographs of different machining voltage: (**a**) ultrasonic vibration assistance; and (**b**) non-ultrasonic vibration assistance.

The pulse voltage was changed from 4.8 to 6.2 V. The rule curve of machined groove width and SEM measurement results are shown in Figure 10 and Figure 11. To ensure no short circuit happens during the machining process, the minimum voltage that can be used without ultrasonic vibration assistance is 5.6 V, and the smallest microgroove width is 144 µm. At the same time, the minimum voltage that can be used with ultrasonic vibration is 4.8 V, and the smallest microgroove width is 138 µm. Because ultrasonic vibration promotes electrolyte circulation and improves the machining environment, smaller voltages can also meet machining needs, and the machining stability is improved. When the voltage increases, the material removal rate and secondary corrosion rate increase, resulting in groove width increases and reduced machining accuracy. The groove surface quality with ultrasonic assistance is higher. The microgroove can be machined with smaller voltage parameters, and the width of the microgroove machined is smaller.

### 3.3. Effect of Pulse Width on Machining Quality

**Figure 12:**
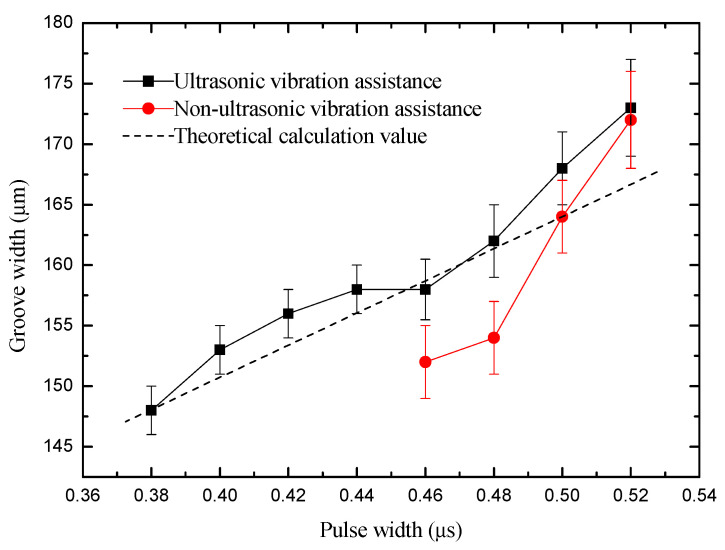
Change of groove width with pulse width.

**Figure 13:**
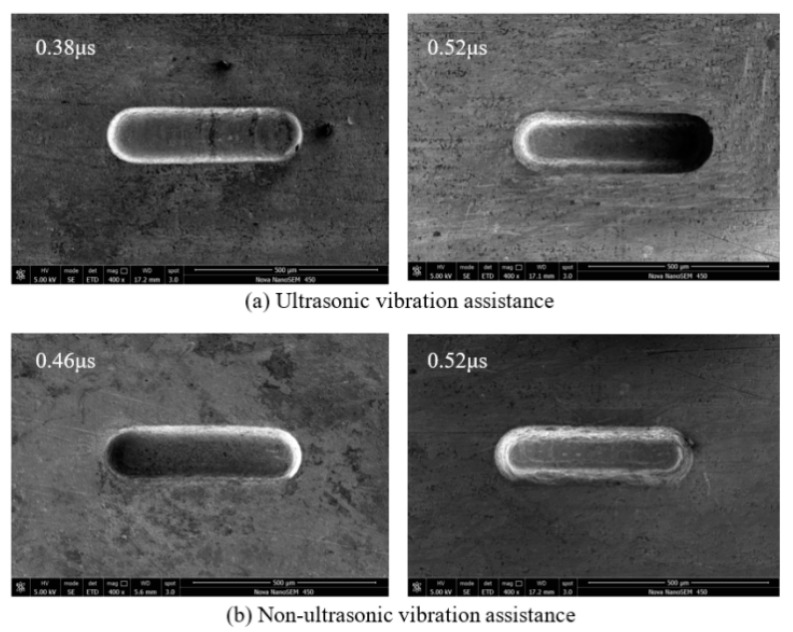
SEM micrographs of different pulse width: (**a**) ultrasonic vibration assistance; and (**b**) non-ultrasonic vibration assistance.

After considering the above voltage range, the applied voltage was fixed to 5.6 V. The pulse width was changed from 0.38 to 0.52 µs. The rule curve of machined groove width and SEM measurement results under different pulse width are shown in Figure 12 and Figure 13. Under the same machining condition, the minimum pulse width is 0.46 µs and the minimum width of microgroove machined is 152 µm without ultrasonic vibration assistance, whereas they are 0.38 µs and 148 µm, respectively, with ultrasonic vibration. The amount of material removal per unit time increases when using a large pulse width, resulting in poor machining environment. Therefore, to improve the machining precision, a smaller pulse width should be used. Compared with the same machining condition without ultrasonic vibration assistance, the machining precision and surface quality with ultrasonic vibration assistance are better.

### 3.4. Effect of Pulse Period on Machining Quality

**Figure 14:**
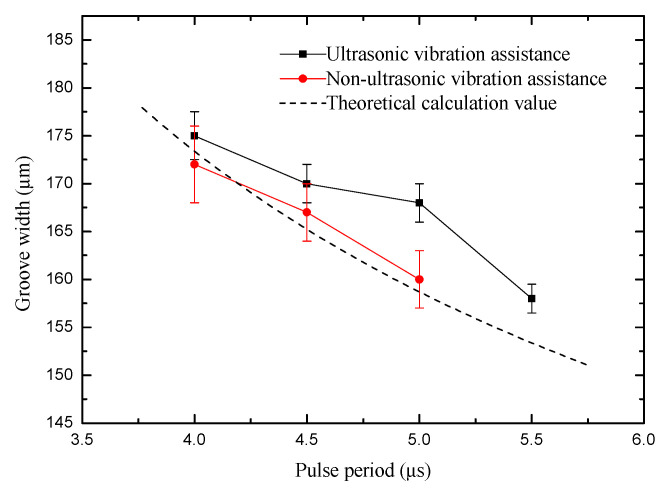
Change of groove width with pulse period.

**Figure 15:**
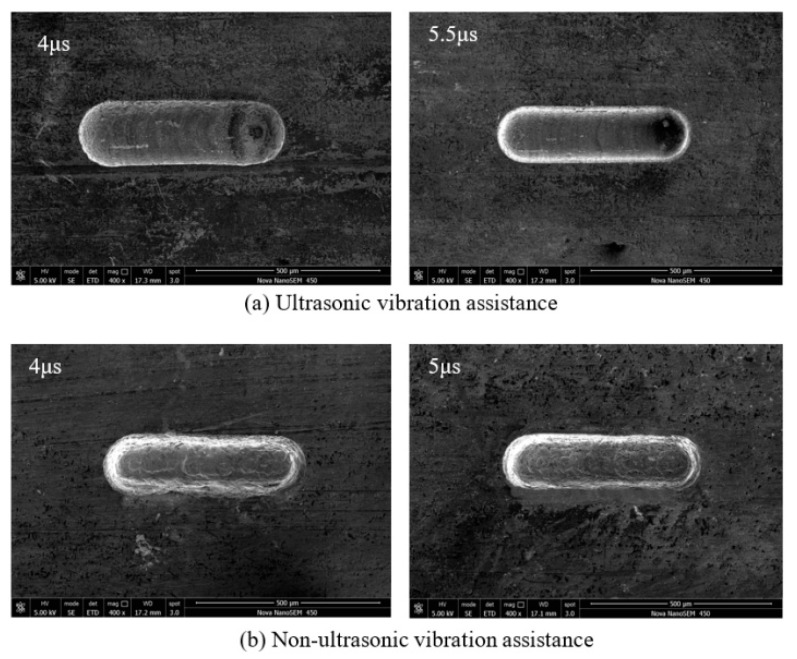
SEM micrographs of different pulse period: (**a**) ultrasonic vibration assistance; and (**b**) non-ultrasonic vibration assistance.

The pulse width was 0.46 µs and the pulse period was changed from 4 to 5.5 µs. The rule curve of machined groove width and SEM measurement results under different pulse period are shown in Figure 14 and Figure 15. Under the premise of ensuring no short circuit during machining process, the maximum pulse period that can be used without ultrasonic vibration assistance is 5 µs, and the smallest microgroove width is 160 µm. Correspondingly, the maximum pulse period that can be used with ultrasonic vibration is 5.5 µs, and the smallest microgroove width is 158 µm. Because ultrasonic vibration improves the machining stability, microgrooves can also be machined with a larger pulse period. As the pulse period decreases, stray corrosion on both sides of the microgrooves becomes more serious. When the pulse period decreases, total output energy in a certain time increases and energy used for secondary corrosion increases. Thus, stray corrosion is more serious, and the width of microgrooves increases. Owing to the improvement of machining stability by ultrasonic vibration, the microgroove can be machined with smaller power supply parameters and the width of the microgroove machined is smaller.

### 3.5. Effect of Feed Speed on Machining Quality

**Figure 16:**
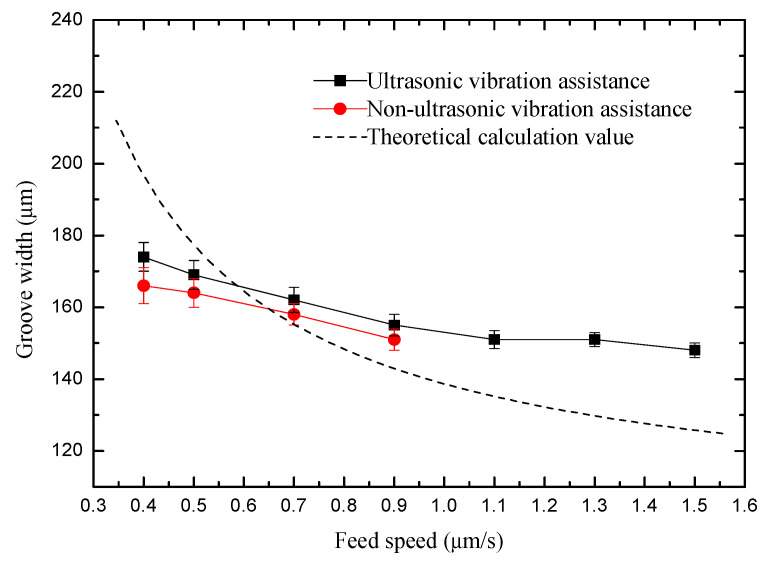
Change of groove width with feed speed.

**Figure 17:**
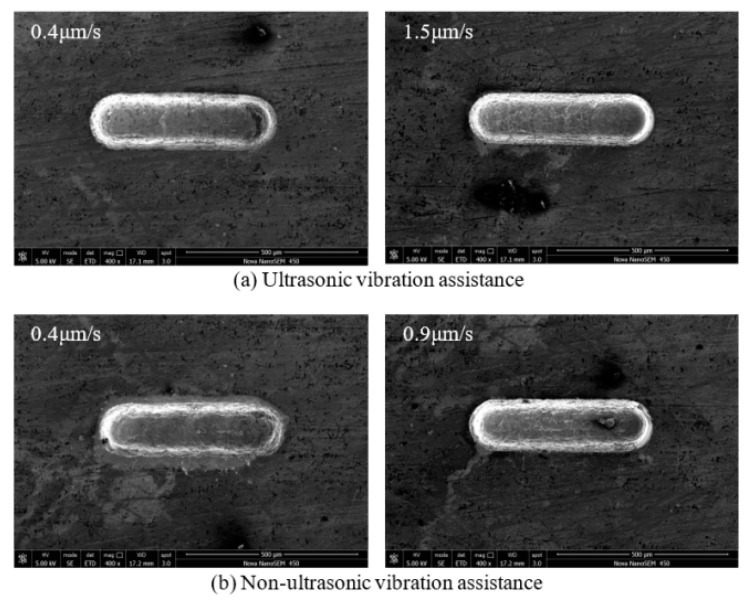
SEM micrographs of different feed speed: (**a**) ultrasonic vibration assistance; and (**b**) non-ultrasonic vibration assistance.

Feed speed represents machining efficiency. The feed speed was changed from 0.4 to 1.5 µm/s. After the experiment above, the voltage, pulse width and pulse period were set to 5.6 V, 0.46 µs and 5 µs, respectively. The rule curve of the machined groove width and SEM measurement results are shown in Figure 16 and Figure 17. Under the same machining parameters, the maximum transverse feed speed without ultrasonic vibration assistance is 0.9 µm/s and the machined minimum groove width is 151 µm, while the transverse feed speed assisted by ultrasonic vibration is 1.5 µm/s and the machined minimum groove width is 147 µm. The machining precision of the microgrooves is improved with the increase of feed speed. This is because the larger feed speed reduces the time of secondary corrosion. At the same time, the surface quality is better than microgrooves with non-ultrasonic assistance.

## 4. Results

To show the advantages of ultrasonic vibration-assisted micro electrochemical milling performance, some two- and three-dimensional microstructures were fabricated under optimized machining parameters.

### 4.1. Two-Dimensional Microstructures

**Figure 18:**
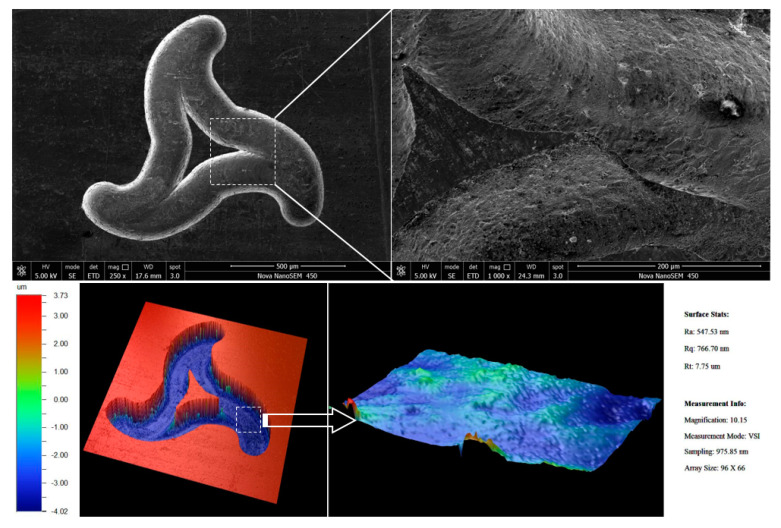
Two-dimensional microstructures.

Experimental parameters were as follows: spindle speed of 12,000 rpm, milling depth of 100 µm, feed speed of 1.2 µm/s, machining voltage of 5.2 V, pulse parameters of 5 µs period and 0.46 µs width and ultrasonic amplitude of 10 µm. Figure 18 shows the machined curve microstructure. The machined surface roughness is about *R*a 0.55 µm.

### 4.2. Three-Dimensional Microstructures

**Figure 19:**
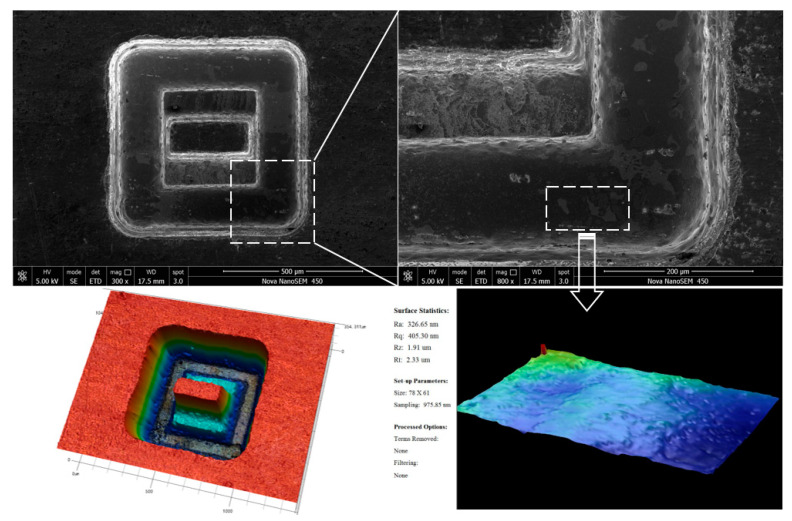
Three-dimensional microstructures.

The three-dimensional microstructure was milled in three layers, each with a milling depth of 100 µm. The machining voltage, pulse width and pulse period were 5.0 V, 0.4 µs and 5 µs, respectively. The ultrasonic amplitude was 10 µm and the feed speed was 1.5 µm/s. The milling depth of the boundary was 300 µm, and the milling depth of intermediate boss was 200 µm. Figure 19 shows the machined complex 3D microstructure and the machined surface roughness is about *R*a 0.33 µm.

## 5. Conclusions

This paper proposes an effective ultrasonic vibration-assisted micro electrochemical milling technology. The following conclusions can be obtained:

(1) The mathematical model of UA-MECM process was established. The trend of theoretical calculation values fit well with the following experimental values. The intrinsic mechanism of this process is still worth exploring.

(2) The ultrasonic vibration can improve the machined surface quality. Machining with a 10- µm-amplitude ultrasonic vibration reduced the surface roughness from *R*a 0.83 to 0.26 µm.

(3) The machining performances of UA-MECM including machining quality and efficiency were proved by experimental results. Under the appropriate ultrasonic conditions, the limit machining voltage was reduced from 5.6 to 4.8 V, the minimum pulse width was reduced from 0.46 to 0.38 µs and the maximum feed rate was increased from 0.9 to 1.5 µm/s.

(4) Two- and three-dimensional microstructures with good surface quality and dimensional accuracy were fabricated using the optimized machining parameters, which shows the superiority of ultrasonic vibration-assisted micro electrochemical milling. It is proved that the proposed UA-MECM technology has potential and broad industrial application prospects.

## Figures and Tables

**Table 1:** The simulation parameters.

Electrode Diameter (µm)	Machining Gap (µm)	Ultrasonic Amplitude (µm)	Vibration Frequency (KHz)	Feed Rate (r/min)
100	50	10	25	12,000

**Table 2:** General machining parameters.

Milling Depth/Length (µm)	Electrode Diameter (µm)	Electrolyte Concentration	Workpiece Material	Ultrasonic Amplitude (µm)
100/400	100	5% NaNO_3_	0Cr18Ni9	0–10
